# Human gastric cancer risk screening: From rat pepsinogen studies to the ABC method

**DOI:** 10.2183/pjab.97.023

**Published:** 2021-10-11

**Authors:** Chie FURIHATA

**Affiliations:** *1 Division of Molecular Target and Gene Therapy Products, National Institute of Health Sciences, Kawasaki, Kanagawa, Japan.; *2 Japan Research Foundation of Prediction, Diagnosis and Therapy for Gastric Cancer, Tokyo, Japan.; *3 School of Science and Engineering, Aoyama Gakuin University, Sagamihara, Kanagawa, Japan.

**Keywords:** rat pepsinogen, human pepsinogen, DNA methylation, *Helicobacter pylori*, human gastric cancer risk screening, ABC method

## Abstract

We examined the development of gastric cancer risk screening, from rat pepsinogen studies in an experimental rat gastric carcinogenesis model induced with *N*-methyl-*N*′-nitro-*N*-nitrosoguanidine (MNNG) and human pepsinogen studies in the 1970s and 1980s to the recent “ABC method” for human gastric cancer risk screening. First, decreased expression or absence of a major pepsinogen isozyme, PG1, was observed in the rat gastric mucosa from the early stages of gastric carcinogenesis to adenocarcinomas following treatment with MNNG. In the 1980s, decreases in PGI in the human gastric mucosa and serum were identified as markers of atrophic gastritis. In the 1990s, other researchers revealed that chronic infection with *Helicobacter pylori* (*Hp*) causes atrophic gastritis and later gastric cancer. In the 2000s, a gastric cancer risk screening method combining assays to detect serum anti-*Hp* IgG antibody and serum PGI and PGII levels, the “ABC method”, was established. Eradication of *Hp* and endoscopic follow-up examination after the ABC method are recommended to prevent gastric cancer.

## Introduction

Gastric cancer remains one of the most common and fatal cancers worldwide, especially among older men. According to GLOBOCAN 2020 data, gastric cancer is the sixth most common neoplasm and the third most deadly cancer, with an estimated 768,793 deaths in 2020.^1)^ In Japan, gastric cancer was the most deadly cancer until 1992,^2)^ and was the second most common neoplasm and the third most deadly cancer in 2019.^3)^ The number of deaths due to gastric cancer in Japan was the highest at 50,680 in 1998,^4)^ and gradually decreased to 43,000 in 2019.^3)^ However, the number of deaths caused by gastric cancer in older men remains high, at up to 350/100,000 person-years at the age of 85.^5)^

Animal models of chemical carcinogenesis have been used for many years in Japan. The first successful chemical carcinogenesis study in the world was conducted by Yamagiwa and Ichikawa in 1915, who generated carcinoma in the ear of a domestic rabbit using coal tar.^6)^ In the 1930s, Sasaki and Yoshida induced liver cancer in rats using *ortho*-aminoazotoluene.^7,8)^ Tumor production in the glandular stomach of rats induced by *N*-methyl-*N*′-nitro-*N*-nitrosoguanidine (MNNG), a known mutagen, was achieved by Sugimura and Fujimura in 1967.^9)^ These findings initiated the study of gastric carcinogenesis in rat stomach using MNNG.

## Experimental rat gastric carcinogenesis studies using pepsinogen as a marker

Around 1970, it was postulated that gastric cancer was induced by chemical carcinogens in food or in the environment, or by chemical substances produced in the body.^10)^ Studies on the stomach were limited, and few suitable markers had been identified. In this era, pepsinogen was shown as a precursor of pepsin, a digestive enzyme, in the stomach.^11)^ Thus, a gastric carcinogenesis study using pepsinogen as a marker in the rat gland stomach mucosa was initiated under the supervision of Sugimura.

### Decrease or absence of PG1 protein in the glandular stomach of rats during gastric carcinogenesis induced by MNNG.

**Figure 1.**
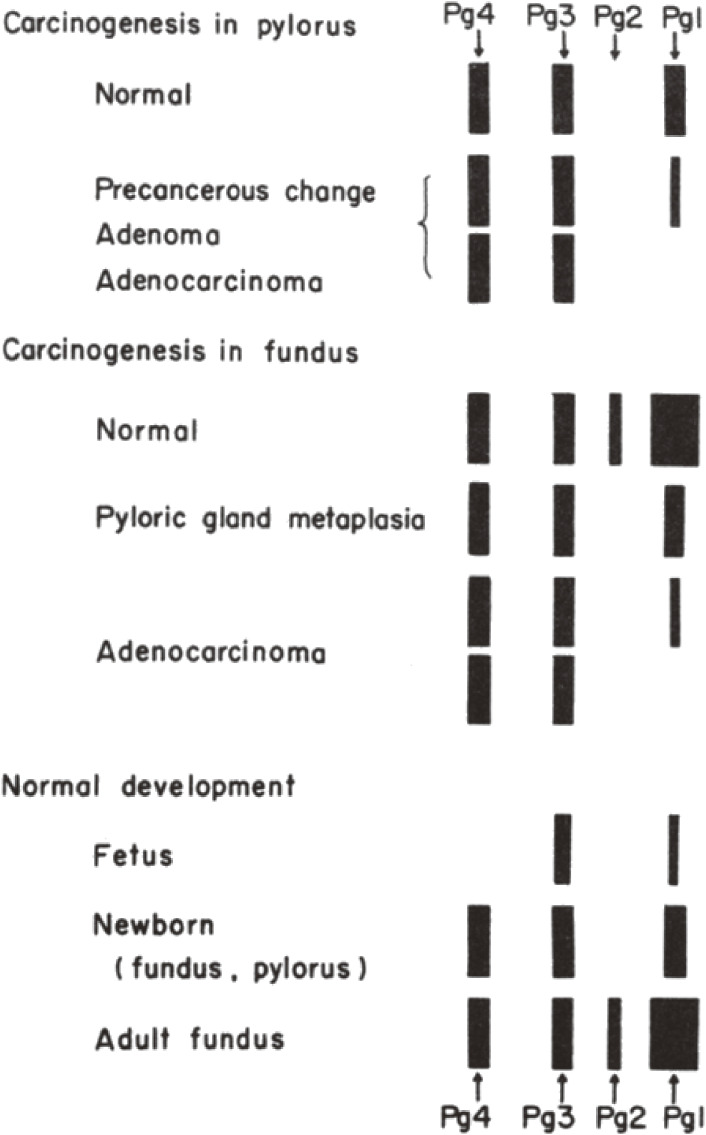
Electropherogram of rat pepsinogens. Changes in pepsinogen isozyme patterns during stomach carcinogenesis and in normal development from fetus to adult. Pepsinogen extracts from samples were run on a 7.5% polyacrylamide gel in 50 mM Tris-acetate buffer (pH 8.2). Then the gel was immersed in a solution of 0.65% hemoglobin in 60 mM HCl, incubated in a humid chamber at 37 ℃, and stained with 1% amido black 10B in 7% acetic acid. Destaining was achieved in 7% acetic acid.^13,14,41,42)^ Bands show relative quantity of pepsin derived from pepsinogen isozymes. Pg1, pepsinogen 1; Pg2, pepsinogen 2; Pg3, pepsinogen 3, Pg4, pepsinogen 4. “Normal” in “Carcinogenesis in pylorus or fundus” means normal pylorus and normal fundus.

In the first notable study of gastric carcinogenesis, decreased or absent expression of a pepsinogen isozyme, PG1, was induced in the gastric mucosa after administration of MNNG to rats.^12–28)^ Downregulated PG1 protein expression was induced in the pyloric mucosa as early as 1 week to 10 months after the administration of MNNG (83 µg/ml) to 8-week-old male Wistar rats, as detected by polyacrylamide gel electrophoresis (Fig. 1). Decreased PG1 and PG2 expression, the latter a minor pepsinogen isozyme, was also observed in the fundic mucosa. Remarkable histopathological changes including atrophic changes were observed in the pyloric mucosa from 8 months after MNNG was administered, and rats showing such unusual histological alterations also had changes in their pepsinogen isozyme pattern.^12–14)^

Other studies showed that downregulated PG1 protein expression induced by MNNG was observed in 28 of 30 well-differentiated adenocarcinomas and two adenomas in Wistar rat stomach^15)^ and in two of two transplantable well-differentiated adenocarcinomas induced by MNNG and 4-nitroquinoline 1-oxide.^15,16)^ Short-term (8 days) administration of MNNG induced DNA damage and heritable permanently reduced PG1 protein in the pyloric mucosa of inbred male Wistar rats. This effect was the response of newly arisen pyloric gland cells that were derived from stem cells modified by MNNG, and these modified stem cells continuously and irreversibly produced altered pyloric gland cells until at least 455 days.^17)^

Furthermore, dose-dependent induction of altered pyloric glands with low PG1 expression in Fischer rat stomach after the administration of MNNG for 5, 8, and 12 weeks was detected using an immunohistochemical assay with anti-PG1 serum. The results suggest that the appearance of pyloric glands with low PG1 expression may be a preneoplastic change in gastric carcinogenesis.^18)^

In another experiment, male F344/Du Crj rats were given drinking water containing 100 pg/ml MNNG for 30 weeks and then normal tap water thereafter, and were examined in weeks 10, 20, 30, 40, 50, and 70. After MNNG treatment, class III mucin-positive pyloric glands with low PG1 protein content in normal-looking pyloric mucosa were identified from week 10, and their number subsequently increased with time. Adenomatous hyperplasias were found from week 30 and adenocarcinomas were observed from week 50. These results suggested that the appearance of pyloric glands with low PG1 expression in normal-looking mucosa might be an immunohistochemically detectable preneoplastic change that precedes morphologically detectable preneoplastic changes in stomach carcinogenesis.^19)^

**Figure 2.**
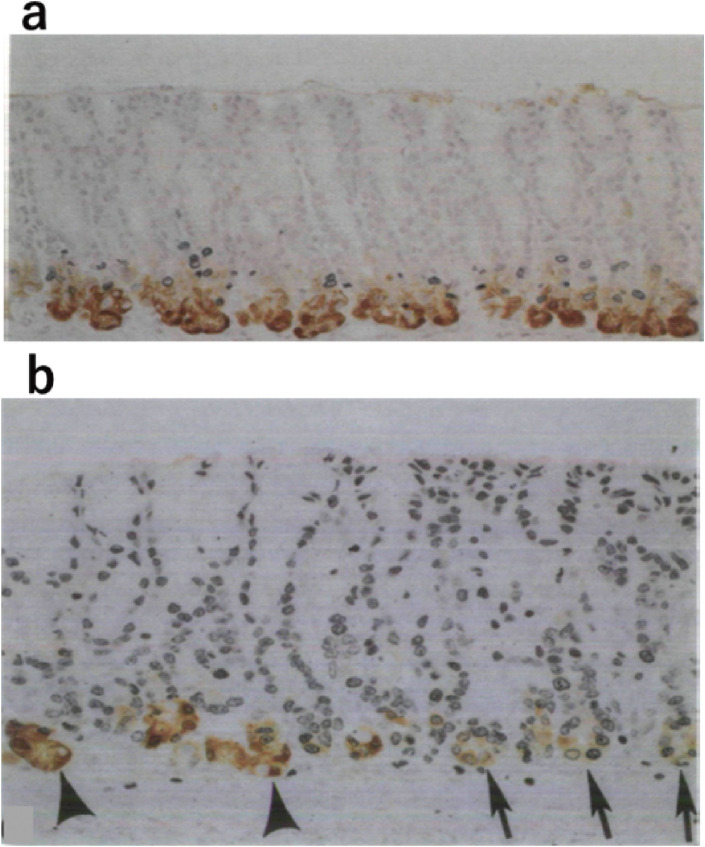
Immunohistochemical staining of cells in PG1-altered pyloric gland (PAPG) with low PG1 protein content. Double immunohistochemical staining for BrdU and PG1 of normal-looking pyloric mucosa treated with MNNG (100 µg/ml MNNG in drinking water) undertaken 7 days after continuous labelling with BrdU. a, normal control. b, almost all cells in PAPG are labelled with BrdU (arrows). However, pyloric gland cells with high PG1 content located in the lower one-third of the pyloric gland (arrowheads) demonstrated no incorporation of BrdU.^20)^

Another study analyzed the kinetics of PG1-altered pyloric gland (PAPG) cells with low PG1 expression using double immunohistochemical staining for bromodeoxyuridine (BrdU) incorporation and PG1 in male WKY/N Crj rats treated with MNNG, as shown in Fig. 2.^20)^ After continuous BrdU administration, the life span of PAPG cells was estimated to be approximately 6–8 days, whereas that of normal pyloric gland cells was approximately 11–13 days. Thus, the data indicated that PAPG cells demonstrated a degree of independence from the pyloric glands with regards to the proliferation kinetics, which suggests that PAPG is a preneoplastic lesion involved in gastric carcinogenesis.

Finally, decreased pepsinogen content and PG1 in the pyloric mucosa of the rat stomach was induced in the pyloric mucosa of Wistar rat stomachs by short-term administration of MNNG and other stomach carcinogens, *N*-ethyl-*N*′-nitro-*N*-nitrosoguanidine and *N*-propyl-*N*′-nitro-*N*-nitrosoguanidine, but not by liver carcinogens, diethylnitrosamine, and dimethylnitrosamine.^21,22)^

### Aberrant methylation of PG1 during gastric carcinogenesis.

It was demonstrated that DNA methylation affects PG1 gene expression in rat glandular stomach mucosa.^29–34)^ Ichinose *et al.* revealed using methylation analysis with *MspI/HpaII* and *HhaI* that there was an inverse correlation between DNA methylation and PG1 gene expression during normal stomach development. There was no detectable PG1 mRNA in either primary or transplanted Wistar–Imamichi rat gastric cancers induced by MNNG. Moreover, the methylation patterns of PG1 were different from those of normal tissues that expressed the gene and those that did not, and no simple correlation was observed between the methylation and expression of PG1.^29)^

In another study, administration of MNNG for 30 weeks to male WKY/NCrj rats induced PAPGs, adenomatous hyperplasias, and well-differentiated adenocarcinomas. Adenomatous hyperplasias and adenocarcinomas all consisted of gastric type cells resembling surface mucous cells or pyloric gland cells with little or no PG1 expression. In MNNG-induced gastric cancers generally lacking PG1, altered PG1 methylation was observed, with both CCGG and GCGC sites being methylated more than normal pyloric mucosa. These results suggest that the altered methylation of PG1 observed in gastric cancers is acquired early in the carcinogenic process, and that progressive changes in methylation occur with tumor development.^30)^

### Intestinal metaplasia is important not as a precancerous lesion but rather as a paracancerous condition in rat gastric carcinogenesis.

Tatematsu *et al.* hypothesized that intestinal metaplasia is important not as a precancerous lesion but rather as a paracancerous condition in various experimental animal gastric carcinogenesis models, including in rats treated with MNNG,^35–38)^ mice with *N*-methyl-*N*-nitrosourea (MNU),^39)^ and Mongolian gerbils with MNU and *Hp*.^40)^

Gastric adenocarcinomas induced by MNNG (50 µg/ml) in Wistar rats were examined using paradoxical concanavalin A staining and biochemical PG assay, which revealed that they were mainly composed of gastric-type tumor cells. Only gastric type cells were observed in 21 of 30 adenomatous hyperplasias and in 19 of 36 well-differentiated adenocarcinomas. The others consisted chiefly of gastric type cells but partly contained intestinal type cells. However, the percentage area of the intestinal type was extremely small, at less than 3.5%. Adenomatous hyperplasias or well-differentiated adenocarcinomas composed only of intestinal type cells were not observed. These findings suggest that neoplastic germ cells produce mainly gastric type cells and sometimes intestinal type cells. Most tumor cells apparently originate from areas of mucosa composed of gastric type cells.^35)^

### Rat pepsinogen isozymes.

In a study examining the expression of pepsinogen, at least four pepsinogen isozymes (PG1, PG2, PG3, and PG4) from rat stomach mucosa were separated by electrophoresis on polyacrylamide gels, and PG1, PG2, PG3, and PG4 were present in the adult fundic mucosa; PG1 was the major PG isozyme in the adult fundic mucosa, as shown in Fig. 1.^13,14,41–43)^ Chief cells and mucous neck cells in the fundic mucosa were found to produce pepsinogen.^41–43)^ PG1, PG3, and PG4 were present in the pyloric mucosa, and PG3 was relatively predominant at all stages after birth, whereby pepsinogen was produced by the pyloric gland cells.^41–46)^

Differentiation of pepsinogen-producing cells was induced in the fundic mucosa but not in the pyloric mucosa of developing rats after birth to 30 days. In the fundic mucosa, increases in the peptic activity of pepsinogen, changes in the molecular species of pepsinogen isozymes separated by electrophoresis, and changes in the morphology of the chief cells indicated that maturation of the chief cells began around 15 days after birth and was complete 25–30 days after birth. In the pyloric mucosa, no changes in the peptic activity or molecular species of pepsinogen or morphology of pyloric gland cell occurred after birth. The isozymes pattern of adult pylorus was the same with newborn pylorus, as shown in Fig. 1.^13,14,42)^ Injection of hydrocortisone or adrenocorticotropic hormone caused a precocious increase in peptide activity in the gastric mucosa in infant Wistar–Imamichi rats.^41)^ The differentiation of pepsinogen-producing cells was additionally studied.^47–50)^

Four pepsinogen isozymes, PG1, PG2, PG3, and PG4 were purified and characterized from the fundic mucosa of male Wistar–Imamichi inbred adult rats.^51)^ The amino acid compositions of these four zymogens differed from each other but resembled those of pepsinogen Cs from various animal species. Activated and purified pepsin 1 from PG1 was a unique pepsin showing remarkable stability in alkaline conditions. It resembles pepsin A with respect to its inhibition by pepstatin and its amino acid composition, but had properties intermediate between those of pepsin A and pepsin C regarding its optimal pH (2.1 to 3.1) with hemoglobin and its activity to N-acetyl-L-phenylalanyl-L-diiodotyrosine. Furthermore, rabbit antiserum prepared against PG1 reacted with PG2 but not with PG3 and PG4.^51)^

During stomach development, a progressive increase in PG1 mRNA that almost coincided with changes in the mucosal pepsinogen level and progressive demethylation after the onset of transcription was observed. Thus, there was an inverse correlation between the methylation and expression of PG1 genes, which suggests a role of DNA methylation in PG1 gene regulation during normal differentiation, although not in its primary role in gene activation.^29)^

### Rat pepsinogen genes and proteins registered with the National Center for Biotechnology Information (NCBI).

Three rat pepsinogen genes, *Pgc*, *Pga5* and *Cym*, are registered with the NCBI. *Pgc* (official full name: progastricsin. gene ID: 24864, also known as *PG1*, location: chromosome 9) is registered as an adult rat pepsinogen gene.^52–54)^ Additionally, *Pga5* (official full name: pepsinogen A5, gene ID: 60372, neonatal zymogen of pepsins, location: chromosome 1)^55,56)^ and *Cym* (official full name: chymosin, gene ID: 56825, location: chromosome 2, major neonatal pepsinogen)^57)^ are registered as neonatal pepsinogen genes.

Three rat pepsinogen proteins (pepsinogen, pepsinogen F protein, and embryonic pepsinogen precursor) are registered in NCBI. Progastricsin (pepsinogen C) (392-amino acid protein, accession: AAA41827) is registered as an adult rat pepsinogen protein.^58)^ Additionally, pepsinogen F protein (pepsinogen 5, group I) (387-amino acid protein, accession: CAB75982)^59)^ and embryonic pepsinogen precursor (prochymosin) (379-amino acid protein, accession: NP_064476)^60)^ are registered as neonate/infant-specific pepsinogens.

Rat pepsinogen C has a similar amino acid composition to PG1 in Ref. 51. Rat pepsinogen F and prochymosin have a different amino acid composition to PG1–4 in Ref. 51.

## Additional studies on rat gastric carcinogenesis

### Induction of various biological responses by MNNG in rat gastric mucosa.

MNNG induces unscheduled DNA synthesis (UDS, a marker of DNA damage),^61,62)^ DNA single-strand scission (DSSS),^62,63)^ c-fos and c-myc oncogene expression,^64)^ immune response gene expression [MHC class II, MHC class II-associated invariant chain, OX-62 (dendritic cell marker) and ED-1 (dendritic cell and macrophage common marker)]^65–67)^ and osteonectin-expressing cells^68)^ in the pyloric mucosa of rat stomach.

### Screening of chemical gastric carcinogens and identification of NaCl as a gastric tumor promoter.

*In vivo* short-term assays for tumor initiation and promotion in the glandular stomach of Fischer rats were undertaken.^69,70)^ UDS and DSSS were used as markers of tumor-initiating activity, and replicative DNA synthesis and ornithine decarboxylase activity were analyzed as markers of tumor-promoting activity.^69–71)^ The possible tumor-initiating and -promoting activities of glyoxyl,^72,73)^ diacetyl,^72)^ 3-diazo-*N*-nitrosobamethan,^74)^ hickory smoke condensate,^75,76)^ omeprazole,^77–79)^ nitrosated *Oroxylum indicum* Vent,^80)^ 2-chloro-4-methylthiobutanoic acid,^81)^ *p*-methylcatechol,^82)^ methylhydroquinone^82)^ and *N*-methyl-*N*-nitrosourea (MNU)^83)^ were examined; however, strong chemical gastric carcinogens in foods or the environment or chemical substances in the body were not identified.

Conversely, NaCl was identified as a gastric tumor promoter by Takahashi and Tatematsu *et al.*^84,85)^ We participated in studies on the possible tumor-promoting activities of NaCl^86–90)^ and other chemicals.^91–98)^

Epidemiologically, tobacco smoking is a known cause of gastric cancer.^99)^ Furthermore, a higher daily intake of alcohol was suggested to be associated with a higher risk of gastric cancer.^100)^

## Experimental mouse gastric carcinogenesis studies using pepsinogen as a marker

PAPGs were also observed in an MNU-induced experimental model of gastric carcinogenesis induced various mouse strains including BALB/c, C3H, C57BL/6N, CBA, CD-1, DBA/2, and db/db diabetic mice.^101–107)^ *Trp53* and *ras* gene mutations were rarely observed in mouse gastric tumors induced by MNU.^108)^

## Gastric carcinogenesis studies using pepsinogen as a marker in human gastric cancer

### Early human serum pepsinogen studies.

Serum pepsinogen was identified at least 100 years ago.^109)^ The relationship between low serum pepsinogen and atrophic gastritis was suggested in at least the 1960s.^110)^ Intensive human pepsinogen studies were initiated by Samloff *et al.* from around 1970, and the presence of pepsinogen isozymes in gastric mucosa, urine, and serum was detected by agar gel electrophoresis.^111–114)^ There are two immunologically distinctive human pepsinogens, group I pepsinogens (PGI) and group II pepsinogens (PGII).^111,112)^ Group I pepsinogens correspond with the main component of acid proteinases, pepsin, in gastric juice, and group II pepsinogens correspond with gastricsin.^113)^

The relationship between low pepsinogen proteins in human gastric mucosa and gastric cancer was reported in the 1970s by Hirsch-Marie *et al.*^115)^ and Furihata *et al.*,^13)^ and the relationship between low serum pepsinogen and gastric cancer was reported around 1980 by Samloff *et al.*^116)^ and Miki *et al.*^117)^

**Figure 3.**
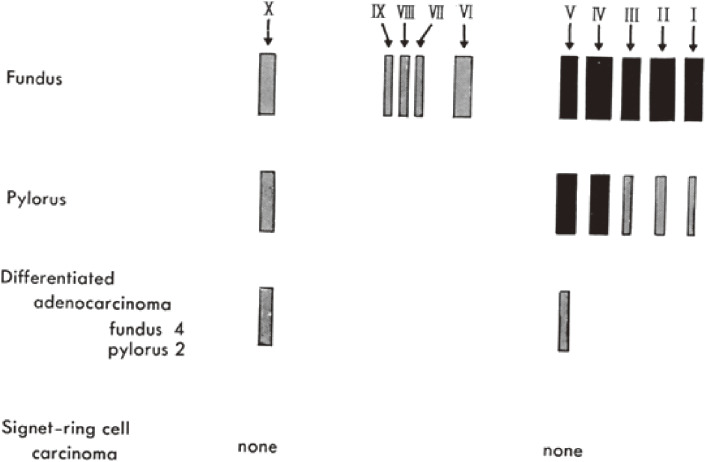
Electropherogram of human pepsinogens. Pepsinogen extracts from samples were run on a 7.5% polyacrylamide gel. After electrophoresis, the gel was treated as described in Fig. 1.^13)^ I, II, III, IV, V, VI, VII, VIII, IX and X are PG1, PG2, PG3, PG4, PG5, PG6, PG7, PG8, PG9 and CD. Band size and color density indicate the relative quantity of pepsin derived from pepsinogen isozyme. PG2 and PG4 are the major pepsinogens in the fundic mucosa and PG5 is the major pepsinogen in the pyloric mucosa.

At least nine pepsinogen isozymes proteins (PG1–9) and a cathepsin D-like acid proteinase (CD) in the fundic mucosa were separated by polyacrylamide gel electrophoresis. In the pyloric mucosa, PG4, PG5, and CD were observed and PG2, PG3, and PG7–9 were occasionally observed, as shown in Fig. 3.^13,118,119)^

### Decreased PGI in human gastric mucosa and serum during gastric carcinogenesis.

**Figure 4.**
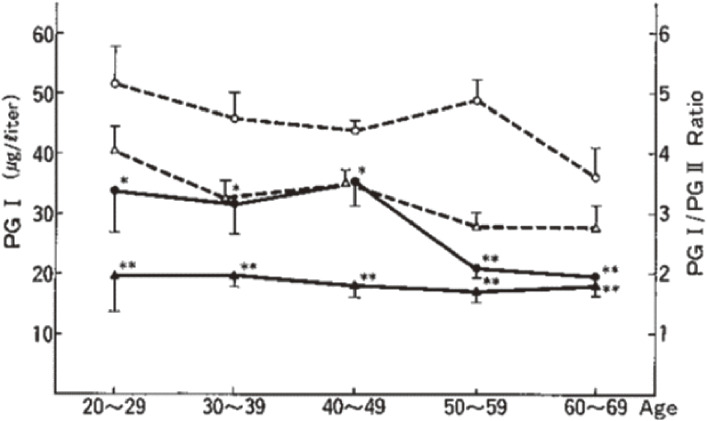
Chronic changes in serum PGI and PGI/PGII ratio in cancer patients and cancer-free-subjects. The number of patients with cancer and cancer-free subjects in each age group were seven and 41 (20–29 years), 14 and 63 (30–39 years), 21 and 85 (40–49 years), 34 and 69 (50–59 years), and 61 and 30 (60–69 years), respectively. Control: ○, PgI; △, PgI/PgII. Cancer: ●, PgI; ▲, PgI/PgII. Vertical lines show the range of standard errors. Differences versus the cancer-free subjects: *, P < 0.05; **, P < 0.001 (Student’s *t*-test).^126)^

To identify serum PGI and PGII, PGI and PGII were purified from gastric mucosa and prepared for radioimmunoassay.^120–122)^ Enzyme-linked immunosorbent assays for serum PGI and PGII were further developed using monoclonal antibodies.^123–125)^ This was the first human serum PGI and PGII assay kit using monoclonal antibodies globally. Chronic gastritis was studied using this kit, and useful biomarkers of atrophic gastritis (serum PGI and PGII and the PGI/PGII ratio) were established.^126,127)^ Serum pepsinogen levels were measured in 137 patients with gastric cancer and compared with those of 288 normal cancer-free subjects. The serum pepsinogen levels of gastric cancer patients, especially PGI and the PGI/PGII ratio were significantly lower than those of normal controls and correlated well with the extent of chronic gastritis associated with the cancerous stomach, as shown in Fig. 4.^126)^ PGI levels gradually decreased, while those of PGII did not decrease, and a stepwise reduction in the PGI/II ratio was closely correlated with the progression from normal gastric mucosa to extensive atrophic gastritis.^127)^ This marker indicates a precancerous change in the stomach, rather being a tumor marker.^128)^ The high sensitivity and specificity of this non-invasive serum test to detect chronic gastritis suggested the possibility of its application to the mass screening of gastric cancer risk. Furthermore, the usefulness of gastric cancer risk screening using the serum pepsinogen test method was demonstrated in multiple studies.^117,129–132)^ In gastric cancer tissues and cancer cell lines, the expression of pepsinogen proteins was decreased or lost, correlating with their production.^118)^

### Altered methylation of pepsinogen genes during human gastric carcinogenesis.

Ichinose *et al.* reported that PGA and PGC genes are hypomethylated in tissues producing PGA and PGC, which suggests a role for DNA methylation in the regulation of their differential expression during normal differentiation.^133,134)^ In gastric cancer tissues and cancer cell lines, no gross structural changes of the pepsinogen genes were observed, but the methylation patterns of the pepsinogen genes were found to be altered in different ways in various cancers. The functional significance of this altered methylation is unknown; however, these results suggest considerable heterogeneity in the methylation patterns of human gastric cancers.^134,135)^ The observed correlation between altered DNA methylation levels and the activity of *Hp*-related gastritis appears to be a relevant molecular mechanism underlying the development of diffuse-type cancer.^136)^ Furthermore, Ushijima *et al.* reported that comprehensive DNA methylation was observed in gastric cancer.^137–140)^

### Is intestinal metaplasia a preneoplastic change in human gastric cancer?

Previously, Correa hypothesized that gastric carcinogenesis occurred in the following sequential stages: chronic gastritis; atrophy; intestinal metaplasia; and dysplasia.^141)^ He speculated that intestinal metaplasia is an essential step in gastric carcinogenesis but this is an ongoing research area.^142,143)^

Tatematsu *et al.*, however, proposed that intestinal metaplasia may not be a preneoplastic change in gastric carcinoma, but rather that intestinal type cells may appear independently in gastric cancer or in the gastric mucosa.^118,119)^ Using a combined histochemical and immunohistochemical approach to detect mucin, they classified surgical specimens of 301 differentiated gastric cancers into three types: gastric epithelial cell (G) type, intestinal epithelial cell (I) type, and mixed gastric and intestinal cell (GI) type, according to the phenotypic differentiation of the component cancer cells. The proportion of G type cancers was 41.4% in early (tumor invasion of mucosa or submucosa) cases, decreasing to 22.2% in advanced (tumor invasion of muscularis propia or deeper) cases, whereas the proportion of I type cancers increased with progressive disease from 23.5% to 31.1% (P < 0.01). Cancer invading the subserosa or deeper included more I type cases and fewer G type cases than cancer limited to the mucosa (P < 0.01). A phenotypic shift from G to I type expression was observed with the progression of human differentiated gastric cancers. Intestinalization may occur independently in cancerous and non-cancerous gastric mucosa.^144,145)^ Recently, a considerable number of studies raised the question as to whether intestinal metaplasia is a direct precursor of gastric cancer or merely a marker of high cancer risk in human gastric cancer.^146,147)^

### Human pepsinogen genes and proteins registered in NCBI.

Pepsinogen A3, pepsinogen A4, and pepsinogen A5 (*PGA3*, *PGA4* and *PGA5*) and progastricsin (*PGC*) are registered in NCBI/Gene.^148–152)^ *PGA3*, *PGA4* and *PGA5* are located on chromosome 11, while *PGC* (also known as *PGII*) is located on chromosome 6.

Regarding pepsinogen proteins, three pepsinogen A (group I) proteins (388-amino acids proteins pepsinogen 3, pepsinogen 4, and pepsinogen 5) are registered in NCBI/Protein.^153–155)^ The amino acid compositions of these three proteins vary by just a few amino acids. In addition to pepsinogen C (progastricsin, 388 amino acids), gastricsin isoform 1 preproprotein (388 amino acids) and gastricsin isoform 2 preproprotein (315 amino acids) are registered in NCBI/Protein.^156,157)^ The amino acid compositions of pepsinogen C and gastricsin isoform 1 preproprotein are identical, while that of gastricsin isoform 2 preproprotein differs after the 217th amino acid. Nonetheless, correlations between the electrophoretic patterns of human pepsinogen isozymes, genes, and proteins registered in NCBI have not been clarified.

## Carcinogenicity of chronic infection with *Hp*

The presence of spiral-shaped microorganisms in the human stomach was described more than 100 years ago by Jaworski.^158)^ *Hp* was first identified in the human stomach in 1982 by Marshall and Warren.^159)^ There is sufficient evidence in humans for the carcinogenicity of chronic infection with *Hp* (Group 1).^160,161)^ Chronic infection with *Hp* causes non-cardiac gastric cancer and low-grade B-cell MALT gastric lymphoma,^160)^ while chronic infection with *Hp* CagA-positive strains is the strongest risk factor of gastric cancer. CagA protein is delivered into gastric epithelial cells via bacterial type IV secretion.^162)^ *Hp* infection is now recognized as the main acquired factor involved in the pathogenesis of peptic ulcer disease and chronic gastritis^163)^ as well as gastric cancer. Furthermore, a relationship between *Hp* and serum pepsinogens in asymptomatic Japanese population was reported.^163,164)^

*Hp* infection occurs during childhood, most commonly before 5 years of age. In Japan, older generations born before around 1950 show a high prevalence of *Hp* infection of approximately 80–90%, decreasing with age to approximately 10% or less in those born in the 1990s, and less than 2% of children born after 2000.^165)^ Once infection is established, it usually lasts for life unless treated.^161)^ *Hp* infection is generally treated with a 1-week regimen of triple therapy consisting of an antisecretory agent and two antibiotics.^166)^ Eradication of *Hp* decreases the severity of gastritis, producing significant changes in serum PG levels, including the serum PGI to PGII ratio.^167)^

## ABC method

Asaka and Miki initiated a study of the relationship between anti-*Hp* IgG antibody and serum pepsinogen in the 1990s.^163,164,168)^ Miki *et al.* proposed a new gastric cancer risk screening method by combining the assays for serum anti-*Hp* IgG antibody and serum pepsinogen level (PGI and PGI/PGII ratio) detection and categorizing the findings into four groups, where PG-negative means normal control PG, and PG-positive indicates that PGI and the PGI/PGII ratio are significantly lower than those of normal controls: A (*Hp*-negative, PG-negative); B (*Hp*-positive, PG-negative); C (*Hp*-positive, PG-positive); and D (*Hp*-negative, PG-positive).^169–172)^

Miki established the ABC method in 2011.^171)^ Subjects were classified into one of four risk groups on the basis of the results of the two serologic tests for anti-*Hp* IgG antibody titers and PGI and PGII levels: Group A [*Hp* (−), PG (−)], infection-free subjects; Group B [*Hp*(+), PG(−)], chronic atrophic gastritis (CAG)-free or mild; Group C [*Hp*(+) PG(+)], CAG; and Group D [*Hp*(−) PG(+)], severe CAG with extensive intestinal metaplasia. Continuous endoscopic follow-up examinations are required to detect the early stages of gastric cancer. Asymptomatic Group A, which accounts for 50–80% of subjects, may be excluded from the secondary endoscopic examination for efficiency. *Hp*-infected subjects should be administered eradication treatment aimed at the prevention of gastric cancer.

## Application of the ABC method in gastric cancer risk screening

Hundreds of studies were reported in various countries on serum anti-*Hp* IgG antibody, serum PG levels, and human gastric cancer^167–185)^ after Miki published the ABC method in 2011.^171)^

Ichinose *et al.* reported a 16-year prospective study of 4,655 healthy asymptomatic subjects (men aged 40–59 years at the start of the study) in Wakayama in Japan, which demonstrated that the combination of *Hp* antibody and serum PG was a good predictive tool of gastric cancer incidence. In subjects with a serologically diagnosed healthy stomach (*Hp*-negative, PG-negative), the cancer incidence rate was low, at 16/100,000 person-years. With the establishment of *Hp* infection and the progression of chronic gastritis, significant stepwise cancer risk elevations were observed in chronic atrophic gastritis (CAG)-free subjects (*Hp*-positive, PG-negative) [hazard ratio (HR) 8.9, 95% confidence interval (CI) 2.7–54.7] to subjects with CAG (*Hp*-positive, PG-positive) (HR 17.7, 95% CI 5.4–108.6), and finally to subjects with metaplastic gastritis (*Hp*-negative, PG-positive) (HR 69.7, 95% CI 13.6–502.9).^174)^ These results clearly indicated that serum levels of PG and *Hp* antibody titers provided indices of cancer development in *Hp*-infected subjects.

Song *et al.* showed that the combination of PGI and *Hp* antibody was useful for predicting gastric cancer in their large Finnish cohort.^180)^ Serum PGI (sPGI) measurements were available for 21,895 Finnish male smokers. In a subset (n = 3,555) with anti-*Hp* serology, groups B, C, and D had increased gastric cancer ORs of 1.79 (95% CI 1.21–2.64), 3.85 (95% CI 2.36–6.28), and 6.35 (95% CI 2.20–18.34), respectively. Low sPGI was associated with increased gastric cancer risk in their cohort. Thus, a single measurement of sPGI along with *Hp* whole-cell measurements and CagA serology provides a potentially useful prediction tool for gastric cancer risk.

The ABC method has been confirmed as a valid approach for gastric cancer risk screening. Leja *et al.* conducted the GISTAR study, which was a multicentric randomized study of *Hp* eradication and pepsinogen testing for the prevention of gastric cancer mortality.^181)^ Furthermore, a statistical study confirmed that the ABC method is useful for the estimation of gastric cancer lifetime cumulative incidence risk and risk of mortality.^182)^ The “Gastric Cancer Checklist” (https://epi.ncc.go.jp/riskcheck/gastric/, in Japanese) of the division of Public Health/Health Science/Hygiene, National Cancer Center Research Institute, Japan, also incorporates the ABC method.

A combination of assays for the presence of serum anti-*Hp* IgG antibody and serum PG concentration can be used to screen for gastric cancer risk. In Japan, serum anti-*Hp* IgG antibody and serum PG assays plus scheduled endoscopy is a cost-effective method for the screening of gastric cancer risk.^183)^

*Hp* eradication can change PG levels and serum *Hp* sero-positivity and *Hp* eradicated individuals should be excluded from the ABC method. Further investigation for these individuals is necessary regarding screening of gastric cancer risk.

## Conclusion

Pepsinogen studies in an experimental rat gastric carcinogenesis model induced by a chemical carcinogen, MNNG, is a good model for the study of human gastric cancer. However, it was recognized in the 1990s that *Hp* infection is the main acquired factor involved in the pathogenesis of peptic ulcer disease and chronic gastritis, alongside gastric cancer. Decreased or absent PG1 protein in the rat gastric mucosa, from the early stage of MNNG-induced gastric carcinogenesis to adenocarcinomas, was identified in the 1970s, and aberrant DNA methylation of PG1 in an experimental gastric carcinogenesis model induced by MNNG was reported in the 1980s. It was proposed that intestinal metaplasia is important not as a precancerous lesion but rather as a paracancerous condition. In the 1980s, it was demonstrated that decreased expression of the major pepsinogen isozyme, PGI, in the human gastric mucosa and serum is a marker of atrophic gastritis, a precancerous lesion of gastric cancer, and the DNA methylation patterns of the pepsinogen genes were found to be altered in different ways in human gastric cancer. It was proposed that intestinal metaplasia may not be a preneoplastic change in gastric carcinoma, but rather that intestinal type cells may appear independently in gastric cancer or in the gastric mucosa. A trial of gastric cancer risk screening using serum PGI and the PGI/PGII ratio was undertaken in the 1980s. In 2011, Miki established the ABC method using a combined assay for serum *Hp* IgG antibody and serum PG levels. The ABC method plus scheduled endoscopy is a cost-effective method of gastric cancer risk screening in Japan, and *Hp*-infected subjects should be administered eradication treatment aimed at the prevention of gastric cancer and the current ABC method may be improved with the progress of future research.

## Non-standard abbreviation list

ABC method: screening of gastritis A, B, C, D
BrdU: bromodeoxyuridine
CD: cathepsin D-like acid proteinase
Cym: chymosin
DSSS: DNA single-strand scission
G type: gastric epithelial cell type
GI type: mixed gastric and intestinal cell type
*Hp*: *Helicobacter pylori*
I type: intestinal epithelial cell type
MNNG: *N*-methyl-*N′*-nitro-*N*-nitrosoguanidine
MNU: *N*-methyl-*N*-nitrosourea
NCBI: National Center for Biotechnology Information
PAPG: PG1-altered pyloric gland
PG: pepsinogen
PGA: pepsinogen A (PGA3, PGA4, and PGA5)
PGC: progastricsin
PGI: groupI pepsinogens
PGII: groupII pepsinogens
UDS: unscheduled DNA synthesis
